# Alendronate-Eluting Biphasic Calcium Phosphate (BCP) Scaffolds Stimulate Osteogenic Differentiation

**DOI:** 10.1155/2015/320713

**Published:** 2015-06-29

**Authors:** Sung Eun Kim, Young-Pil Yun, Deok-Won Lee, Eun Young Kang, Won Jae Jeong, Boram Lee, Myeong Seon Jeong, Hak Jun Kim, Kyeongsoon Park, Hae-Ryong Song

**Affiliations:** ^1^Department of Orthopedic Surgery and Rare Diseases Institute, Korea University Medical College, Guro Hospital, No. 80, Guro-dong, Guro-gu, Seoul 152-703, Republic of Korea; ^2^Department of Oral and Maxillofacial Surgery, Kyung Hee University Dental Hospital at Gangdong, Kyung Hee University, 892 Dongnam-ro, Gangdong-gu, Seoul 134-727, Republic of Korea; ^3^Department of Molecular & Cell Biology, University of California, Berkeley, 142 LSA, No. 3200, Berkeley, CA 94720-3200, USA; ^4^Disease Research Center, Korea Basic Science Institute, Gangwondaehakgil-1, Chuncheon, Gangwon-do 200-701, Republic of Korea

## Abstract

Biphasic calcium phosphate (BCP) scaffolds have been widely used in orthopedic and dental fields as osteoconductive bone substitutes. However, BCP scaffolds are not satisfactory for the stimulation of osteogenic differentiation and maturation. To enhance osteogenic differentiation, we prepared alendronate- (ALN-) eluting BCP scaffolds. The coating of ALN on BCP scaffolds was confirmed by scanning electron microscopy (FE-SEM), energy-dispersive X-ray spectroscopy (EDS), and attenuated total reflectance-Fourier transform infrared spectroscopy (ATR-FTIR). An *in vitro* release study showed that release of ALN from ALN-eluting BCP scaffolds was sustained for up to 28 days. *In vitro* results revealed that MG-63 cells grown on ALN-eluting BCP scaffolds exhibited increased ALP activity and calcium deposition and upregulated gene expression of Runx2, ALP, OCN, and OPN compared with the BCP scaffold alone. Therefore, this study suggests that ALN-eluting BCP scaffolds have the potential to effectively stimulate osteogenic differentiation.

## 1. Introduction

Bone tissues possess the intrinsic capacity for regeneration as part of the repair process in response to injury, skeletal development, or continuous remodeling throughout adult life [1]. Bone regeneration is a complex and well-orchestrated process of biological events of bone induction and conduction to optimize skeletal repair and restore skeletal function [1, 2]. In complex clinical conditions, however, bone regeneration is required in large quantities for the repair of large bone defects created by trauma, infection, tumor resection, and skeletal abnormalities [3]. To stimulate or augment bone regeneration of large bone defects, bone grafting such as autologous bone grafts and allografts is a commonly performed surgical procedure [4, 5]. However, these bone grafts have several limitations such as limited supply, harvest site morbidity, additional blood loss, and inflammatory responses [4, 5].

Bone graft substitutes have been developed as alternatives to autologous or allogenic bone grafts. Such substitutes (i.e., collagen, hydroxyapatite [Hap], *β*-tricalcium phosphate [*β*-TCP] calcium-phosphate cements, and glass ceramics) are synthetic or natural biomaterial scaffolds that promote the migration, proliferation, and differentiation of bone cells for bone regeneration [3, 6, 7]. Biphasic calcium phosphate (BCP) ceramics consist of a mixture of hydroxyapatite (HAp) and beta-tricalcium phosphate (*β*-TCP). BCP exhibits good biocompatibility and bone conduction performance in hard tissue repair because of their similarity to the minerals in human bone and their outstanding bioactivity [8, 9]. As BCP is resorbed* in vivo* it releases calcium and phosphate ions into the microenvironment of the implant site; these ions can be used for new bone formation [10]. Also, the presence of porosity and a bioactive surface on BCP scaffolds facilitates cell attachment, proliferation, and differentiation and consequently favors increased bone formation [11–13]. However, due to lack of inherent osteoinductivity, limited new bone formation occurs after osteoconduction is achieved. Thus, conventional BCP requires the additional use of osteoinductive biomolecules such as bone morphogenic proteins (BMPs) for enhanced osteogenesis [9, 14, 15].

To promote bone formation of scaffolds, the use of bioactive molecules such as growth factors is very attractive. Among various bioactive growth factors, including bone morphogenic proteins (BMPs), basic fibroblast growth factor (bFGF), platelet-derived growth factor (PDGF), and vascular endothelial growth factor (VEGF), BMPs are well known to induce osteogenic differentiation of mesenchymal stem cells and to stimulate ectopic bone formation [16–18]. However, there are problems associated with its clinical application such as the large doses of BMPs required (2.1–12.0 mg), a short half-life* in vivo*, and high cost [19–21]. To overcome these shortcomings, investigators have been looking for alternative drugs. Bisphosphonates have been widely used as potent osteoinductive factors for the treatment of osteoporosis and several bone diseases such as bone metastasis, hypercalcemia of malignancy, and Paget's disease [22]. As one of the most commonly used bisphosphonates, ALN suppresses osteoclast activity by inhibiting the activity of farnesyl pyrophosphate synthetase [23]. Recent studies reported that ALN enhances the osteogenesis of osteoblasts, bone marrow mesenchymal stem cells (BMSCs), and adipose-derived stem cells (ADSCs) [24–26]. ALN can also upregulate mRNA expression during osteogenic differentiation* in vitro*, including that of BMP-2, type I collagen, osteocalcin, and osteopontin [27, 28]. Unlike most drugs, ALN has strong negative charge on the two phosphonate moieties. Thus, its oral bioavailability is very low, averaging only 0.6~0.7% under fasting conditions. To overcome the limitation of low bioavailability, recent studies reported that the local delivery systems including ALN-containing titanium, microspheres, nanoparticles, and chitosan scaffolds could improve osteogenesis of osteoblasts for bone regeneration [27, 29, 30].

In this study, we developed ALN-eluting BCP (ALN/BCP) scaffolds as local delivery system for improving bone formation. Based on the fact that ALN has a high binding affinity to the bone mineral hydroxyapatite (HAp) [31, 32], we fabricated ALN/BCP scaffolds by simply mixing BCP scaffolds with ALN. We hypothesized that ALN/BCP scaffolds may enhance the drug efficacy by achieving the sustained delivery over a period of time as well as enhancing the osteogenesis of osteoblasts compared to BCP alone. In this study, we aimed to investigate whether ALN/BCP scaffolds can effectively improve* in vitro* osteoblast activity and osteogenic differentiation and to demonstrate whether ALN/BCP scaffolds have great potential for bone regeneration.

## 2. Materials and Methods

### 2.1. Materials

Biphasic calcium phosphate substrates (BCP; HAp = 60%, TCP = 40%; diameter × length = 3 × 8 mm, cylinder type) were kindly donated from OssGen Corporation (Gyeongbuk, Korea). Alendronate was obtained from Samjin Pharmaceutical Corporation (Seoul, Korea). Dulbecco's Modified Eagle's Medium (DMEM), phosphate buffer saline (PBS), fetal bovine serum (FBS), and 1% antibiotics (100 U/mM penicillin and 0.1 mg/mL streptomycin) were purchased from Life Technologies Corporation (Grand Island, NY, USA). 2-(*N*-Morpholino) ethanesulfonic acid (MES) was supplied by Sigma Chemical Co. (St. Louis, MO, USA).

### 2.2. Preparation of Alendronate- (ALN-) Coated BCP Substrates

To prepare ALN-eluting BCP substrates, BCP substrates were immersed in 0.1 M MES buffer (pH 5.6) containing ALN (0.1 or 1 mg) and gently shaken for 24 h. The ALN-coated BCP substrates were collected, washed with distilled water (DW), and dried in a vacuum for 1 day.

### 2.3. Characterization of BCP and ALN-Eluting BCP Substrates

Surface morphologies and elemental compositions of BCP and ALN-coated BCP substrates were evaluated by a variable-pressure field emission scanning electron microscopy (VP-FE-SEM; SUPRA-55VP, Carl Zeiss, Germany) at the Korea Basic Science Institute Chencheon Center. The chemical distribution mapping and compositions of BCP and ALN-coated BCP substrates were determined using an energy-dispersive X-ray spectrometer (EDS). ATR-FTIR (attenuated total reflectance-Fourier transform infrared spectroscopy, Avatar 360, Nicolet Instrument Corp., Madison, WI, USA) spectra had a resolution of 4 cm^−1^ between 2000 and 500 cm^−1^. A commercially available powder was used to obtain the ATR-FTIR spectrum of ALN.

### 2.4. *In Vitro* Release Study of ALN from ALN-Eluting BCP Substrates

To assess ALN release from ALN (0.1 mg)/BCP and ALN (1 mg)/BCP, each substrate was soaked in a 15 mL conical tube with 1 mL PBS. The tubes were then incubated at 37°C with shaking at 100 rpm. At predetermined time points of 1, 3, 5, and 10 h and 1, 3, 5, 7, 14, 21, and 28 days, supernatants were collected and replaced with fresh PBS. The collected supernatants were stored in a deep freezer at −20°C before analysis. The released ALN was determined using a UV/Vis spectrophotometer (DU-530, Beckman Coulter) at a wavelength of 293 nm with a complex of alendronate and standard iron (III) chloride solution.

### 2.5. Cell Morphology

For* in vitro* cell studies, MG-63 cells which were isolated from human bone tissue were used because MG-63 cells are popular cells to demonstrate the osteogenic effects of drugs, peptides, or proteins in/on various substrates. MG-63 cells were maintained with DMEM supplemented including 10% FBS, 50 *μ*g/mL ascorbic acid, 10 nM dexamethasone, 10 mM *β*-glycerophosphate, 100 U/mL penicillin, and 100 *μ*g/mL streptomycin.

To confirm the morphologies of MG-63 cells (osteoblast-like cells) grown on BCP and ALN-eluting BCP substrates, the cells (1 × 10^4^ cells/100 *μ*L) were carefully seeded on the surface of BCP and ALN-eluting BCP substrates in a 24-well tissue culture plate, incubated for 30 min at 37°C, and added to 1 mL of DMEM supplemented with 10% FBS and 1% antibiotics in the presence of 50 *μ*g/mL ascorbic acid, 10 nM dexamethasone, and 10 mM *β*-glycerophosphate. After 24 hours of incubation, the cells on substrates were fixed in 3.7% (v/v) formaldehyde for 30 min. For SEM analysis, cells on substrates were further fixed with 1% osmium tetroxide, dehydrated in a graded series of ethanol/distilled water ranging from 50% to 100% in steps of 10% for 10 minutes each, and then lyophilized for 1 day. The cell morphology was examined with SEM (VP-FE-SEM, SUPRA-VP55, Carl Zeiss). For F-actin staining, the fixed cells were immersed in cytoskeleton buffer (5 × 10^−3^ M NaCl, 150 × 10^−3^ M MgCl_2_, 5 × 10^−4^ M Tris-base, and 0.5% Triton X-100) for 5 min at 4°C followed by blocking buffer (5% FBS, 0.1% Tween-20, and 0.02% sodium azide in PBS) for 30 min at 37°C. The cells on substrates were stained with rhodamine-phalloidin (1 : 200) and DAPI (1 : 10,000), and cell morphology was observed under a confocal laser scanning microscope (LSM700, Zeiss, Oberkochen, Germany).

### 2.6. Alkaline Phosphatase (ALP) Activity

To evaluate early osteogenic differentiation, MG-63 cells were seeded on BCP, ALN (0.1 mg)/BCP, and ALN (1 mg)/BCP substrates in a 24-well tissue culture plate at a concentration of 1 × 10^5^ cells/substrate and incubated for up to 14 days in DMEM supplemented with 10% FBS and 1% antibiotics in the presence of 50 *μ*g/mL ascorbic acid, 10 nM dexamethasone, and 10 mM *β*-glycerophosphate. At predetermined time points of 3, 7, 10, and 14 days, the cells were lysed using 1x RIPA (radioimmunoprecipitation assay) buffer [50 mM Tris–HCl, pH 7.4, 150 mM NaCl, 0.25% deoxycholic acid, 1% tergitol-type-40 (NP-40), and 1 mM ethylenediaminetetraacetic acid (EDTA) including protease and phosphatase inhibitors (1 mM phenylmethylsulfonyl fluoride (PMSF), 1 mM sodium orthovanadate, 1 mM sodium fluoride, 1 *μ*g/mL aprotinin, 1 *μ*g/mL leupeptin, and 1 *μ*g/mL pepstatin)]. The cell lysates were centrifuged at 13,500 rpm for 3 min at 4°C. The supernatants were incubated with* p*-nitrophenyl phosphate solution for 30 min at 37°C. The reaction was stopped by addition of 500 *μ*L of 1 N NaOH. ALP activity was determined by measuring the conversion of* p*-nitrophenyl phosphate to* p*-nitrophenol [33]. Optical density was determined using a microplate reader (Bio-Rad, Hercules, CA, USA) at a wavelength of 405 nm.

### 2.7. Calcium Content

To assess late osteogenic differentiation, MG-63 cells were seeded at a concentration of 1 × 10^5^ cells/mL on BCP, ALN (0.1 mg)/BCP, and ALN (1 mg)/BCP substrates in a 24-well tissue culture plate. After 21 days of culture, the cells were washed with PBS, treated with 0.5 N HCl, and centrifuged at 13,500 rpm for 1 min. The resulting supernatant was used for calcium deposition measurement using a QuantiChrom Calcium Assay Kit (DICA-500, BioAssay Systems, Hayward, CA, USA) according to the manufacturer's instructions. The amount of calcium produced was determined at 612 nm using a microplate reader.

### 2.8. Gene Expression

To determine mRNA expression of the osteogenic differentiation markers runt related transcription factor (Runx2), ALP, osteocalcin (OCN), and osteopontin (OPN), we performed real-time PCR. MG-63 cells (1 × 10^5^ cells/mL) were seeded on BCP, ALN (0.1 mg)/BCP, and ALN (1 mg)/BCP substrates in a 24-well tissue culture plate. After 7 and 21 days of culture, cDNA was synthesized with 1 *μ*g total RNA and oligo (dT) primer using the Superscript First-Strand Synthesis System (Bioneer Inc., Daejeon, South Korea) according to the manufacturer's instructions. The following oligonucleotide primers were used for real-time PCR: Runx-2, (F) 5′-ATG GCA TCA AAC AGC CTC TTC AGC A-3′, (R) 5′-CGT GGG TTC TGA GGC GGG ACA CC-3′; ALP, (F) 5′-GTG GAA GGA GGC AGA ATT GAC CA-3′, (R) 5′-AGG CCC ATT GCC ATA CAG GAT GG-3′; OCN, (F) 5′-TGA GAG CCC TCA CAC TCC TC-3′, (R) 5′-ACC TTT GCT GGA CTC TGC AC-3′; OPN, (F) 5′-GAG GGC TTG GTT GTC AGC-3′, (R) 5′-CAA TTC TCA TGG TAG TGA GTT TTC C-3′; GAPDH, (F) 5′-ACT TTG TCA AGC TCA TTT CC-3′, and (R) 5′-TGC AGC GAA CTT TAT TGA TG-3′. PCR amplification and detection were carried out on an ABI7300 Real-Time Thermal Cycler (Applied Biosystems, Foster, CA, USA) with the DyNAmo SYBR Green qPCR Kit (Finnzymes, Espoo, Finland). The relative mRNA expression levels of Runx2, ALP, OCN, and OPN were normalized to that of GAPDH. All results were confirmed by repeating the experiment three times.

### 2.9. Statistical Analysis

Quantitative data are presented as means ± standard deviation, and comparisons were carried out using one-way ANOVA (Systat Software Inc., Chicago, IL, USA). Differences were considered statistically significant at ^*∗*^ *P* < 0.05.

## 3. Results

### 3.1. Characterization of BCP and Modified BCP Scaffolds

**Figure 1:**
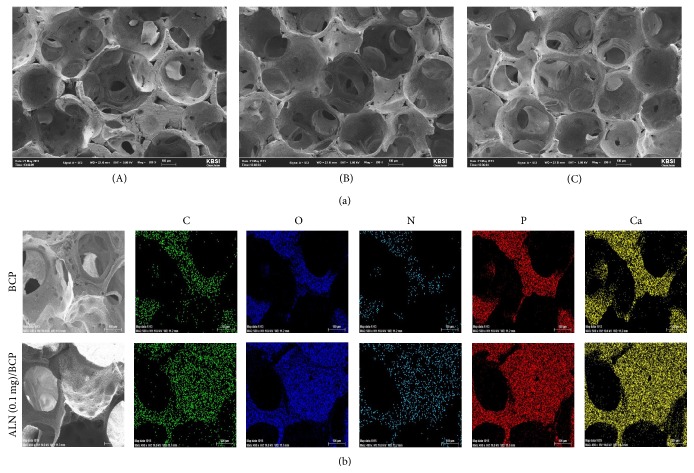
(a) Scanning electron microscope (SEM) images of (A) BCP, (B) ALN (0.1 mg)/BCP, and (C) ALN (1 mg)/BCP scaffolds. (b) The representative chemical mapping images of BCP and ALN (0.1 mg)/BCP scaffolds.

**Figure 2:**
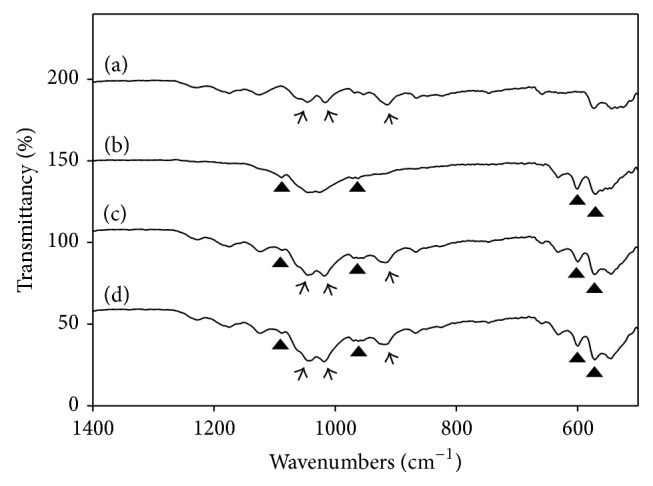
Attenuated total Fourier-transform infrared spectroscopy (ATR-FTIR) spectra of (a) ALN, (b) BCP, (c) ALN (0.1 mg)/BCP, and (d) ALN (1 mg)/BCP scaffolds. The characteristic peaks of alendronate sodium appear at 926 cm^−1^ for the hydroxyl group and at 1020 cm^−1^ and 1050 cm^−1^ for P=O (indicated by the arrows), and BCP appears at 940–1120 cm^−1^, 603 cm^−1^, and 566 cm^−1^ for orthophosphate (PO_4_) (indicated by the black triangles). These experiments were repeated three times.

The surface morphologies of BCP and ALN-BCP scaffolds were investigated by SEM. In SEM images, the surfaces of BCP and ALN (0.1 mg)/BCP and ALN (1 mg)/BCP scaffolds showed similar morphologies, with open and round-shaped pores ranging in size from 100 to 300 *μ*m (Figure 1(a), (A)–(C)). To confirm the successful preparation of ALN-eluting BCP scaffolds, the chemical distribution (i.e., C, N, O, P, and Ca) mapping images of BCP and ALN (1 mg)/BCP scaffolds were obtained by EDS analysis. As shown in representative mapping images of BCP and ALN (0.1 mg)/BCP, colors for C (green), O (blue), N (light blue), and P (red) on ALN (0.1 mg)/BCP were evenly distributed on the surface of BCP scaffolds (Figure 1(b)). The surface elemental chemical compositions (i.e., C, N, O, P, and Ca) of BCP and ALN-BCP scaffolds were also determined with EDS. In particular, successful loading of ALN on the surface of BCP scaffolds was confirmed by an increase in the N content. As the amount of added ALN increased, N content increased from 0% for BCP, 4.60% for ALN (0.1 mg)/BCP, and 8.03% for ALN (1 mg)/BCP scaffolds (Table 1). The loading of ALN on the surface of BCP scaffolds was also confirmed with ATR-FTIR. As shown in Figure 2, the characteristic absorption peaks of orthophosphate (PO_4_) were observed at 940–1120 cm^−1^ and 566 cm^−1^ on BCP and the two ALN/BCP scaffolds. As the amount of added ALN increased, the characteristic peaks for alendronates were observed at 926 cm^−1^ (hydroxyl groups) and 1020 cm^−1^ and 1050 cm^−1^ (P=O stretch) and their peak intensities increased.

### 3.2. *In Vitro* ALN Release from ALN/BCP Scaffolds

**Figure 3:**
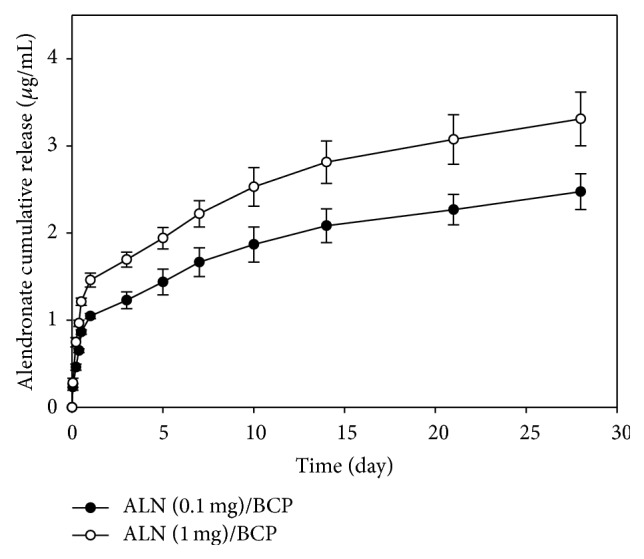
*In vitro* release profiles of ALN from ALN (0.1 mg)/BCP and ALN (1 mg)/BCP scaffolds. These experiments were repeated three times.

Figure 3 shows the release profiles of ALN from ALN (0.1 mg)/BCP and ALN (1 mg)/BCP scaffolds. Sustained release of ALN from ALN (0.1 mg)/BCP and ALN (1 mg)/BCP scaffolds was observed for up to 28 days. On the first day, 1.05 ± 0.03 *μ*g and 1.46 ± 0.08 *μ*g of ALN were released from ALN (0.1 mg)/BCP and ALN (1 mg)/BCP scaffolds, respectively. Subsequently, ALN was slowly released from the ALN/BCP scaffolds. By day 28, 2.48 ± 0.21 *μ*g and 3.31 ± 0.31 *μ*g of ALN were released from ALN (0.1 mg)/BCP and ALN (1 mg)/BCP scaffolds, respectively.

### 3.3. Cell Morphology Study

**Figure 4:**
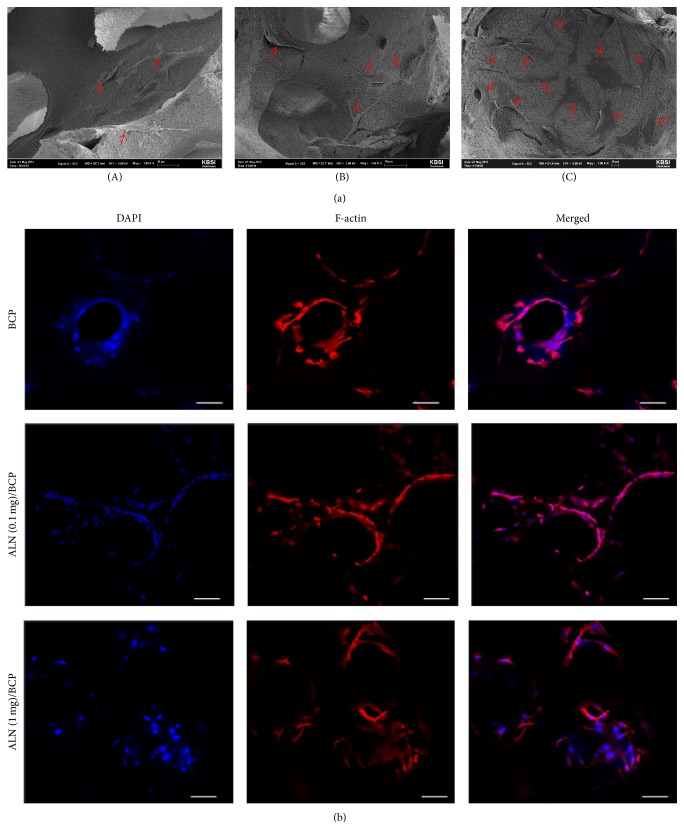
(a) SEM images of MG-63 cells cultured on (A) BCP, (B) ALN (0.1 mg)/BCP, and (C) ALN (1 mg)/BCP scaffolds. (b) F-actin staining of MG-63 cells cultured on BCP, ALN (0.1 mg)/BCP, and ALN (1 mg)/BCP after 24 hours of incubation. Bar: 80 *μ*m. These experiments were repeated three times.

Figure 4 shows the cell morphology of MG-63 cells cultured on BCP and ALN/BCP scaffolds. As shown in Figure 4(a), the MG-63 cells adhered on the surface of BCP and ALN/BCP scaffolds after 24 hours of incubation. The adhered cells were spread on the surface of the scaffolds, and the number of adhered cells increased with an increase in the amount of ALN coating the BCP scaffolds (Figure 4(b)).

### 3.4. *In Vitro* Osteogenic Differentiation Study

**Figure 5:**
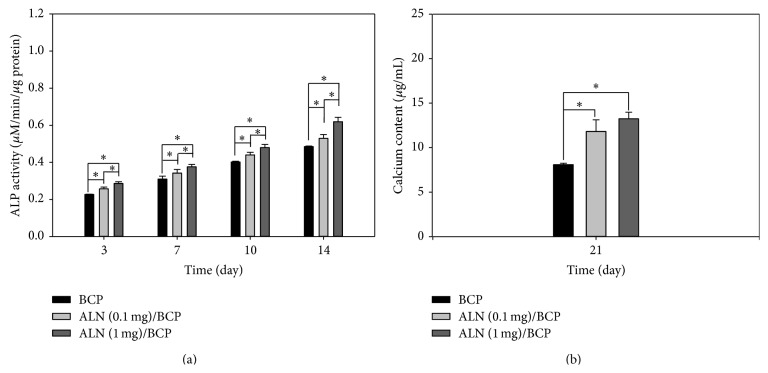
(a) ALP activity of MG-63 cells cultured on BCP, ALN (0.1 mg)/BCP, and ALN (1 mg)/BCP scaffolds after culture for 3, 7, 10, and 14 days (^*∗*^ *P* < 0.05) and (b) calcium deposition by MG-63 cells cultured on BCP, ALN (0.1 mg)/BCP, and ALN (1 mg)/BCP after culture for 21 days (^*∗*^ *P* < 0.05). The error bars represent mean ± SD (*n* = 5). These experiments were repeated three times.

Figure 5(a) shows the ALP activity of MG-63 cells cultured on BCP, ALN (0.1 mg)/BCP, and ALN (1 mg)/BCP scaffolds at 3, 7, 10, and 14 days. The ALP activity of MG-63 cells grown on all scaffolds gradually increased with incubation time up to 14 days. Also, as the coating amount of ALN on BCP scaffolds increased, the ALP activity of MG-63 cells also increased: ALP activity of ALN (1 mg)/BCP > ALN (0.1 mg)/BCP > BCP. Furthermore, the ALP activity of MG-63 cells cultured on ALN (1 mg)/BCP scaffolds was significantly higher than that of cells on ALN (0.1 mg)/BCP and BCP at all incubation time points (^*∗*^ *P* < 0.05). The amount of calcium deposition was determined after culture of MG-63 cells for 21 days on BCP, ALN (0.1 mg)/BCP, and ALN (1 mg)/BCP scaffolds (Figure 5(b)). The amount of deposited calcium was 8.08 ± 0.18 for BCP, 11.81 ± 1.32 for ALN (0.1 mg)/BCP, and 13.24 ± 0.74 for ALN (1 mg)/BCP, respectively. Calcium deposition by MG-63 cells cultured on ALN/BCP scaffolds was significantly higher than that on BCP scaffolds (^*∗*^ *P* < 0.05). However, the amount of calcium deposited by MG-63 cells cultured on the two ALN/BCP scaffolds was not significantly different after 21 days of culture.

### 3.5. *In Vitro* Expression of Osteogenic Genes

**Figure 6:**
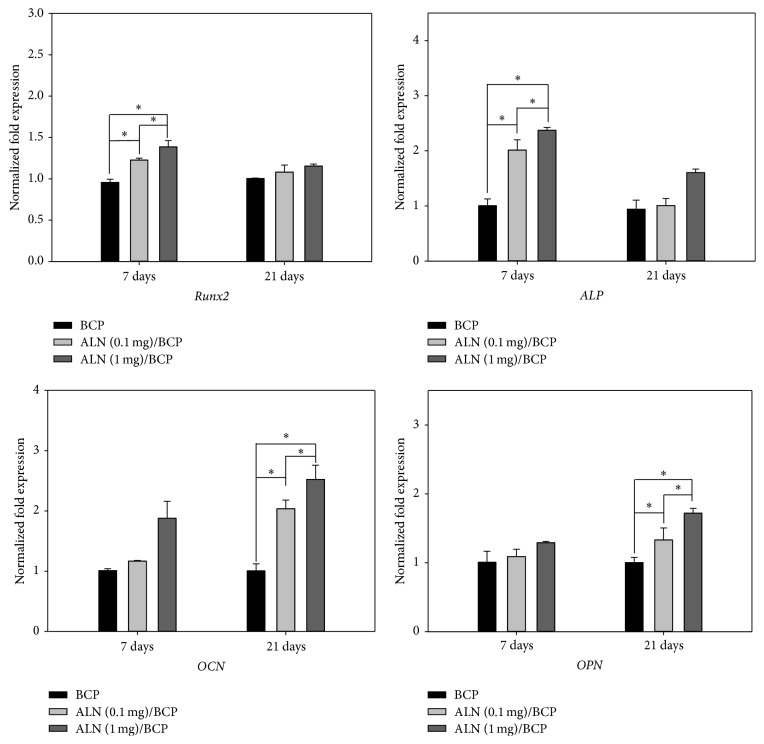
Real-time PCR analysis of mRNA gene expression of Runx2, alkaline phosphatase (ALP), osteocalcin (OCN), and osteopontin (OPN) of MG-63 cells grown on BCP, ALN (0.1 mg)/BCP, and ALN (1 mg)/BCP scaffolds after incubation for 7 and 21 days, respectively, (^*∗*^ *P* < 0.05). The error bars represent mean ± SD (*n* = 5). These experiments were repeated three times.

To further confirm osteogenic differentiation of MG-63 cells, expression of the osteogenic genes Runx2, ALP, OCN, and OPN in MG-63 cells cultured on BCP, ALN (0.1 mg)/BCP, and ALN (1 mg)/BCP scaffolds was evaluated by real-time PCR (Figure 6). At 7 and 21 days, Runx2, ALP, OCN, and OPN mRNA levels in MG-63 cells grown on ALN/BCP scaffolds were higher than those in cells grown on BCP scaffolds. In particular, Runx2 and ALP mRNA levels in MG-63 cells grown on ALN/BCP were significantly higher than those in cells grown on BCP scaffolds at 7 days (^*∗*^ *P* < 0.05). However, there were no significant differences in Runx2 and ALP mRNA levels between BCP and ALN/BCP scaffolds at 21 days. On the other hand, there were no significant differences in OCN and OPN mRNA levels between BCP and ALN/BCP scaffolds at 7 days. At 21 days, however, OCN and OPN mRNA levels in cells grown on ALN/BCP scaffolds were markedly higher than those in cells grown on BCP scaffolds (^*∗*^ *P* < 0.05).

## 4. Discussion

In bone tissue engineering, bone graft substitutes have been used as alternatives to autologous or allogenic bone grafts. Among the choices available, BCP substitutes are considered the most promising as osteoconductive bone substitutes for bone regeneration because they provide a framework for vascular and cellular infiltration and consequently favor new bone formation [11–13] and overcome critical limitations such as expense, limited supply, additional trauma (in the case of autografts), and risk of disease transmission (in the case of allografts) [34]. However, BCP scaffolds do not stimulate osteogenic differentiation of osteoblast-like cells or mesenchymal stem cells. Therefore, in this study, we prepared ALN-eluting BCP scaffolds and evaluated whether they can stimulate osteogenic differentiation of osteoblast-like cells.

Bisphosphonate drugs have hydroxyl groups on the P-C-P structure (hydroxyl methylene bisphosphonate) and show binding affinity to calcium phosphate of bone mineral [31, 32, 35]. Based on the binding affinity of bisphosphonate drugs to bone mineral, we fabricated ALN-eluting BCP scaffolds by simply mixing BCP scaffolds and ALN. Once ALN is mixed with BCP scaffolds, ALN anions are coordinated with calcium cations of the calcium phosphate phase in BCP scaffolds through a bidentate chelation [29]. Significant differences in surface morphologies between BCP and ALN-eluting BCP scaffolds were not observed because immobilization of small-molecular weight ALN might be hard to observe on the surface of subcentimeter-sized BCP scaffolds. The successful loading of ALN on BCP scaffolds was confirmed with EDS and ATR-FTIR, which showed an increase in N content and peak intensities for hydroxyl and P=O groups of ALN on BCP scaffolds. ALN-eluting BCP scaffolds showed sustained release over 28 days, after a burst release during the initial 24 h. The initial burst release might be ascribed to ALN bound on the surface of BCP scaffolds, and the sustained release kinetics might be induced by calcium phosphate dissolution from BCP scaffolds [29].

In the bone tissue regeneration study, initial cell adhesion on the scaffolds is one of the critical factors for cell differentiation [36]. SEM and F-actin staining results showed that the number of adherent MG-63 cells on ALN-eluting BCP scaffolds increased compared to that on BCP scaffolds. This result indicates that the released ALN from ALN/BCP scaffolds might promote favorable cell functions such as cell adhesion on ALN/BCP scaffolds, consistent with our previous study [37]. The enhanced adhesion of osteoblast-like cells by scaffolds also affects three principal osteoblast differentiation processes (proliferation, extracellular matrix maturation, and mineralization) [38]. During these osteogenic differentiation processes, each distinctive stage can be characterized by the expression of distinctive osteogenic markers. As a key component of bone matrix vesicles, ALP is an early indicator of immature osteoblast activity and osteogenic differentiation [36, 39, 40]. After osteogenic differentiation, osteoblastic cells begin to secrete a mineral matrix leading to calcium deposition, a marker for mature osteoblasts [36, 39, 40]. As the most promising bone graft substitutes, BCP scaffolds have been widely studied for bone tissue regeneration. However, BCP scaffolds still have the challenges of insufficient osteogenic differentiation. Compared with BCP scaffolds, ALN-eluting BCP scaffolds significantly enhanced ALP activity and calcium deposition by MG-63 cells in a dose-dependent manner, implying that the released ALN from BCP scaffolds stimulates osteogenic differentiation. Consistent with recent studies, this study also demonstrated that the delivery of ALN using BCP scaffold systems stimulates osteogenic activity and differentiation of osteoblast-like cells for bone tissue regeneration [28, 37].

Only cells that have differentiated to a mature osteoblast phenotype express osteogenic markers such as ALP, Runx2, OCN, and OPN [41, 42]. ALP is observed on the cell surface and in matrix vesicles during osteogenic differentiation. Runx2 plays a crucial role and regulates skeletogenesis and bone formation during the early stages of embryogenesis [43]. OCN and OPN are extracellular matrix proteins and are thought to be important in modulating mineralization. It is well known that ALP and Runx2 are early genes among the osteoblast markers, whereas OCN and OPN are late genes [42, 44–46]. To further confirm the stimulation of osteogenic differentiation by ALN-eluting BCP scaffolds, we examined the gene expression of multiple osteogenic markers including ALP, Runx2, OCN, and OPN. In the BCP scaffold group, expression of these four genes was not increased during the period of cell culture. ALN-eluting BCP scaffolds significantly increased ALP and Runx2 gene expression compared to BCP-only scaffolds at 7 days. However, ALP and Runx2 mRNA levels were downregulated at 21 days, possibly as a result of the switch in cellular processes to mineralization steps [36]. Also, ALN-eluting BCP scaffolds markedly enhanced the gene expression of OCN and OPN in a dose-dependent manner at 21 days. Taken together, these results demonstrate that ALN-eluting BCP scaffolds can deliver ALN for a long period and are sufficient to stimulate osteogenic differentiation. We expect that long-term local delivery of ALN using BCP scaffolds will have great potential for new bone formation at sites of bone defects.

## 5. Conclusions

We fabricated ALN-eluting BCP scaffolds by simple mixing of ALN and BCP scaffolds and evaluated whether they can stimulate osteogenic differentiation of osteoblast-like cells. ALN-eluting BCP scaffolds showed sustained release of ALN for 28 days. Compared to BCP scaffolds, ALN-eluting BCP scaffolds significantly enhanced ALP activity and calcium deposition by osteoblast-like cells. In addition, they markedly increased gene expression levels of osteoblast markers such as ALP, Runx2, OCN, and OPN in a dose-dependent manner. We demonstrated that ALN-eluting BCP scaffolds effectively stimulate osteogenic differentiation and have great potential for bone regeneration.

## Figures and Tables

**Table 1:** Surface elemental composition of BCP and ALN/BCP scaffolds.

Substrate	C (%)	O (%)	P (%)	Ca (%)	N (%)
BCP	10.41	56.24	10.0	23.35	0.0
ALN (0.1 mg)/BCP	22.86	53.60	4.02	14.92	4.60
ALN (1 mg)/BCP	14.80	50.78	5.72	20.68	8.03
